# Phage vB_KpnM_NB cocktail synergizing with amikacin in inhibiting persister cells of *Klebsiella pneumoniae*

**DOI:** 10.1128/aem.01367-26

**Published:** 2026-09-03

**Authors:** Bingqing Xu, Mengye Ma, Yiping Wang, Xingyu Li, Jieting Pan, Yi Jin, Ruiqin He, Lijian Ding, Liming Jiang

**Affiliations:** 1 Department of Emergency Medicine, The Affiliated Yangming Hospital of Ningbo University (Yuyao People’s Hospital), Ningbo University 47862, Ningbo, Zhejiang, China; 2 Ningbo Key Laboratory of Malignant Tumor Immunodiagnosis and Immunotherapy, Health Science Center, Ningbo University 47862, Ningbo, Zhejiang, China; 3 School of Basic Medical Sciences, Health Science Center, Ningbo University 47862, Ningbo, Zhejiang, China; 4 Department of Clinical Laboratory, The Affiliated People's Hospital of Ningbo University 47862, Ningbo, Zhejiang, China; 5 Ningbo Key Laboratory of Precision Treatment and Translation Research for Critical Care, Ningbo, Zhejiang, China; 6 School of Pharmacy, Health Science Center, Ningbo University 47862, Ningbo, Zhejiang, China; 7 School of Pharmacy, Changsha Medical University 216793, Changsha, China; University of Nebraska-Lincoln, Lincoln, Nebraska, USA

**Keywords:** phage cocktail, persister cells, *Klebsiella pneumoniae*, synergistic effect, biofilm

## Abstract

The emergence of multidrug-resistant *Klebsiella pneumoniae* (KPN) and antibiotic-tolerant persister cells poses a significant challenge to existing anti-infection therapies. Given the urgent need for sustainable alternatives to antibiotics, phage cocktails are emerging as a promising alternative to control *K. pneumoniae* infections. We isolated three lytic phages vB_KpnM_NB (1–3) from Ningbo environmental samples, classified them into the Drexlerviridae family, and determined the biological characteristics of two representative phages. Genomic analysis confirmed that these phages are closely related and lack resistance and virulence genes, ensuring biosafety. Subsequently, a stable KPN persister model was established using amikacin, with a biphasic killing pattern observed during treatment. At a multiplicity of infection of 10, the phage cocktail eliminated 99.00% of persister cells, while individual phages were less effective. The phage cocktail also inhibited persister-derived biofilm formation, showing improved results when combined with amikacin. This combination significantly reduced capsule polysaccharide production in persisters, weakening the outer membrane barrier. These findings demonstrate that the phage cocktail-amikacin combination effectively targets planktonic cells, persister cells, and biofilms, providing a promising strategy against persisters and recurrent *K. pneumoniae* infections.

**IMPORTANCE:**

This study fills the critical gap in understanding how phage cocktails synergize with amikacin against *K. pneumoniae* persister cells. By constructing a highly specific phage vB_KpnM_NB cocktail, establishing a stable persister model, and performing *in vitro* bactericidal and biofilm assays, we demonstrate that the cocktail effectively eliminates planktonic cells, persisters, and biofilms. We clarify the core synergistic mechanism: inhibiting capsular polysaccharide synthesis, improving phage adsorption, and disrupting the bacterial outer membrane barrier. These findings provide experimental evidence for the prevention and control of multidrug-resistant and carbapenem-resistant *K. pneumoniae* persister infections, establishing a safe and effective phage-antibiotic combination therapy. The results are crucial for addressing antibiotic tolerance and controlling chronic, recurrent infections. They hold significant theoretical and translational value for the treatment of refractory infections in clinical settings and offer new insights into the development of novel antimicrobial strategies.

## INTRODUCTION

Antimicrobial resistance has become an increasingly serious global health threat (1). *K. pneumoniae* (KPN), a major gram-negative pathogen, is widely distributed on mucosal surfaces of animals and in environmental reservoirs (e.g., water and soil) and commonly colonizes the human gastrointestinal tract (2, 3). It can cause a variety of infections, including respiratory infections, liver abscesses, bloodstream infections, and urogenital infections. Its pathogenicity is closely associated with key virulence factors (4–6). In China, *K. pneumoniae* accounts for approximately 11.90% of pathogens isolated from ventilator-associated pneumonia (VAP) and intensive care unit (ICU)-acquired pneumonia (2). According to data from the China Antimicrobial Surveillance Network (CHINET, 2022), *K. pneumoniae* ranks second among clinical isolates nationwide, with a detection rate of 14%, following *Escherichia coli* (7). There is no doubt that such high rates of incidence and prevalence of infections impose a substantial burden on healthcare systems (8).

Antibiotics have long been the cornerstone of bacterial infection treatment. However, their extensive and often inappropriate use has led to a continuous increase in antibiotic resistance in *K. pneumoniae*. In particular, the emergence of carbapenem-resistant *K. pneumoniae* (CRKP) has become a major clinical challenge (9, 10). CHINET data indicate that the resistance rate of *K. pneumoniae* to carbapenems has increased dramatically from 3% in 2005 to 30% in 2023 (11). The rise of multidrug-resistant (MDR) and even pan-drug-resistant strains has rendered many conventional antibiotics ineffective (12). A meta-analysis reported a mortality rate of 42.14% in patients infected with CRKP compared to 21.16% in those infected with carbapenem-sensitive strains (CSKP) (13). Although strengthened antibiotic stewardship policies in China have slightly reduced CRKP prevalence in recent years, the resistance problem remains unresolved (7).

Furthermore, in addition to resistance, antibiotic-induced persister cells represent another major obstacle in infection treatment. First described by Joseph Bigger in 1944, persister cells are a subpopulation of bacteria that survive antibiotic treatment without acquiring genetic resistance, distinguishing this subgroup of bacteria that survive antimicrobial therapy from drug-resistant mutants (14). Instead, they enter a dormant or non-growing state by downregulating metabolic and biosynthetic activities. Due to reduced transcriptional and translational activity, as well as diminished proton motive force, persister cells exhibit high tolerance to multiple antibiotics (15–17). Under antibiotic exposure, susceptible cells are rapidly eliminated, whereas persisters exhibit a markedly reduced death rate, resulting in a characteristic biphasic killing curve. Once the antibiotic pressure is removed, persister cells can resume growth, serving as a reservoir for infection relapse and microbial “rejuvenation” (18, 19). Persister phenotypes have been widely identified in major pathogens, including *K. pneumoniae*, *E. coli*, and *Staphylococcus aureus* (20–22). Notably, clinical studies have shown that *K. pneumoniae* persisters can persist in sputum samples even after standard decontamination procedures, contributing to chronic and recurrent infections (23).

Conventional antibiotic therapy is often ineffective against persister cells and may even promote their formation, further exacerbating resistance development. Therefore, alternative therapeutic strategies are urgently needed (24). Phages, which specifically infect and lyse bacteria without toxicity to humans, have been approved by the U.S. Food and Drug Administration (FDA) for use in the food industry (25, 26). Phage cocktails, composed of multiple phages with complementary characteristics, have emerged as a promising antibacterial strategy. Tomoyoshi Kaneko et al. isolated 28 phages infecting *E. coli*, evaluated their lytic activity and other characteristics, and clustered the phages based on these properties. They proposed the necessity of combining phages with different physiological characteristics to create a phage cocktail that can effectively lyse bacteria over the long term and inhibit the emergence of drug-resistant strains (27). Researchers from Stanford University in the United States identified “complementary groups” of phages using non-redundant receptors through screening protocols, generating effective broad-spectrum phage cocktails. They created three phage-antibiotic combinations, each demonstrating efficacy against ≥96% of 153 clinical isolates of *Pseudomonas aeruginosa*, and exhibited comparable potency in *in vivo* wound infection models (28). This indicates that phage cocktail therapy has great potential in the treatment of drug-resistant bacterial infections.

Despite these advances, studies on the inhibition of *K. pneumoniae* persister cells by phage cocktails, either alone or in synergy with antibiotics, remain limited, and the underlying mechanisms are not yet fully understood. In this study, *K. pneumoniae*-specific phages vB_KpnM_NB were isolated from river and sewage samples in Ningbo, China. After systematic characterization, a phage cocktail was constructed, and its inhibitory effects on planktonic cells, persister cells, and biofilms of *K. pneumoniae* were investigated, along with the underlying mechanisms. Our results suggest that the phage cocktail combined with amikacin significantly inhibits *K. pneumoniae* persisters, demonstrating effective suppression both in biofilms and capsule polysaccharide (CPS) production. This work aims to provide a theoretical basis for developing novel therapeutic strategies against *K. pneumoniae* persister infections and offers new insights into combating antimicrobial resistance.

## MATERIALS AND METHODS

### Bacterial strains and culture conditions

*K. pneumoniae* XU1 was sourced from the Affiliated Hospital of Ningbo University in Zhejiang Province, China, as a host for phage isolation. The strain was subjected to 16S rRNA gene sequencing, and the results showed high similarity to *K. pneumoniae* strain GDFK0406 (accession number: CP167461). Further identification confirmed that the strain belongs to ST11 and capsular type K2. All bacterial strains were cultured in 37°C Luria-Bertani (LB) broth or LB agar plates (BD Difco, MD, USA). The original culture of *K. pneumoniae* XU1 was preserved in −80°C freezers with 20% (vol/vol) glycerol.

### Isolation and purification of phage vB_KpnM_NB

Phages vB_KpnM_NB (1–3) were isolated from river water and residential sewage samples collected in Ningbo, Zhejiang Province, China. Samples were transported to the laboratory at ambient temperature within 2 h and stored at 4°C prior to use. The isolation procedure was adapted from previous studies with minor modifications (29–31). Briefly, 10 g of each sample was mixed with 20 mL sterile phosphate-buffered saline (PBS) and incubated at room temperature with shaking at 200 rpm for 2 h. The mixture was then centrifuged (4,500 × *g*, 4°C, 10 min), and the supernatant was filtered through a 0.22 μm polyethersulfone (PES) membrane to remove bacterial debris. Subsequently, 15 mL of the filtrate was added to 40 mL LB broth containing a 1:100 dilution of overnight *K. pneumoniae* culture and incubated at 37°C for 48 h. After incubation, the culture was centrifuged (8,000 × *g*, 15 min), and the supernatant was filtered as described above. The filtrate was serially diluted (10-fold), mixed with 5 mL molten 0.45% LB soft agar containing log-phase *K. pneumoniae* (2 × 10^8^ CFU/mL), and immediately poured onto LB agar plates. Plates were incubated at 37°C for 12 h to allow plaque formation. Individual plaques with uniform morphology were picked using sterile pipette tips and transferred into 5 mL LB broth. The purification procedure was repeated three times until pure phage isolates were obtained.

### Transmission electron microscopy (TEM) of phages

The morphology of phage vB_KpnM_NB was examined using TEM (32). A diluted phage suspension (4 × 10^8^ PFU/mL) was deposited onto carbon-coated Formvar copper grids for 10 min and negatively stained with 2% uranyl acetate. Phage particles were visualized using a Philips EM 300 TEM at an accelerating voltage of 80 kV. The capsid diameter and tail length were measured to determine the average particle size.

### Phage genome sequencing and bioinformatics analysis

The high-titer phage lysate was collected, and genomic DNA was extracted from the phages using the Rapid Nucleic Acid Isolation Kit (BioPerfectus, SDKF60101). The quality of the extracted DNA was assessed by agarose gel electrophoresis and NanoDrop spectrophotometer, and samples that met the quality criteria were submitted for sequencing. Phage genome sequencing was performed by the Shanghai Institute of Biomedical Technology using Illumina next-generation sequencing. Briefly, 100 ng of phage DNA was fragmented to approximately 400 bp using a Covaris S2 system (Covaris, USA). DNA libraries were constructed using the NEXTflex DNA Sequencing Kit compatible with the Biomek FXp platform (Bio Scientific, USA). Raw sequencing reads were quality-trimmed using Trimmomatic v0.39, and genome assembly was performed using Newbler software. Open reading frames (ORFs) were predicted using GeneMarkS and further annotated by BLAST (https://blast.ncbi.nlm.nih.gov/Blast.cgi) searches against the NCBI database. Functional annotation was also conducted using the RASTServer (https://rast.nmpdr.org/). The online software tRNAscan-SE 2.0 (https://lowelab.ucsc.edu/tRNAscan-SE/) was utilized to predict the tRNA genes contained in the phage genome. The phage-related taxonomic assignments were confirmed in accordance with the guidelines established by the International Committee on Taxonomy of Viruses (http://www.ictvonline.org/). All annotation results were integrated to obtain complete genome information, which was subsequently submitted to the NCBI database for accession number assignment. ORFs were classified based on predicted protein functions. Genome visualization, including functional annotation maps and GC content analysis, was performed using the Proksee online platform (https://proksee.ca/). Genome annotation was performed using the Prokka software. A phylogenetic tree of the three phages was constructed using molecular evolutionary genetics analysis software (MEGA 10.0).

### Phage stability test and optimal infection multiplicity (MOI)

For thermal stability assays, 1 mL of phage suspension (10^8^ PFU/mL) was incubated at 4, 25, 37, 42, 50, 60, 70, and 90°C for 1 h. For pH stability, buffer solutions with pH values ranging from 3 to 11 were prepared using HCl or NaOH. Then, 0.99 mL of phage suspension (10^8^ PFU/mL) was mixed with 0.99 mL of the adjusted buffer and incubated at room temperature for 1 h. MOI was defined as the ratio of infectious phage particles to host bacterial cells. Phage and bacterial suspensions were mixed at MOIs of 0.0001, 0.001, 0.01, 0.1, 1, and 10, followed by incubation at 37°C with shaking at 220 rpm for 12 h. After incubation, samples were centrifuged (9,000 × *g*, 4°C, 10 min) and filtered through a 0.22 μm membrane to remove bacterial cells. Phage titers were determined using the double-layer agar method, and all experiments were performed in triplicate.

### Adsorption assay and one-step growth curve

To determine the adsorption rate, phage vB_KpnM_NB (1 × 10^8^ PFU/mL) was mixed with log-phase *K*. pneumoniae at an MOI of 0.1 and incubated at 37°C. Samples were collected at 5 min intervals, filtered through a 0.22 μm membrane to remove bacteria, and determined the phage titer in the filtrate. The one-step growth curve was performed with minor modifications based on previously reported methods (33). Briefly, *K. pneumoniae* was cultured to the exponential phase with the optical density at 600 nm (OD_600_) reaching 1.0. Phage was added at an MOI of 1 and allowed to adsorb. The mixture was centrifuged twice (8,000 rpm, 2 min, 4°C) to remove unadsorbed phages, and the pellet was resuspended in 25 mL fresh LB broth. The culture was incubated at 37°C for 150 min, and samples were collected every 10 min. Each sample (300 μL) was centrifuged (10,000 × *g*, 2 min, 4°C), and the supernatant was used to determine phage titers. All experiments were conducted in triplicate.

### Lytic capacity *in vitro*

Phages were co-cultured with bacteria at the exponential growth phase at different multiplicities of infection (MOIs), including 0.1, 1, and 10. The cultures were incubated at 37°C with shaking at 200 rpm. The blank control group contained only LB broth, while the negative control group included bacteria without phages. OD_600_ of the samples was measured at 2, 4, and 8 h.

### Spot test and efficiency of plating

The host range of the phages was determined using the spot test method, following previously reported procedures (34). A total of nine *K. pneumoniae* strains and nine other bacterial strains (*E. coli*, *Enterobacter cloacae*, *P. aeruginosa*, and *Vibrio parahaemolyticus*) were used, and nine types of phages (vB_KpnM_NB1, vB_KpnM_NB2, vB_KpnM_NB3, ZRP-2, ZRP-3, and Sg-1P) were all stored in the laboratory. ZRP-2, ZRP-3, and Sg-1P were isolated from wastewater samples collected from the sewage pool of the People’s Hospital affiliated with Ningbo University. In brief, log-phase bacterial cultures were mixed with molten LB agar and spread onto agar plates. Serially diluted phage suspensions (2 μL) were spotted onto the bacterial lawns and incubated at 37°C overnight. The presence of clear lysis zones indicated susceptibility of the bacterial strain to the phage, whereas the absence of plaques indicated resistance. Subsequently, for all bacterial strains that were shown to be lysed in spot assays, the efficiency of productive infection defined as the efficiency of plating (EOP) was further evaluated using the double-layer agar plaque assay. Briefly, all tested bacterial strains that exhibited clear lytic zones in spot tests were cultured overnight at 37°C with shaking to obtain late-logarithmic-phase bacterial suspensions (1 × 10^8^ CFU/mL). Phage stocks were serially diluted, and the double-layer plaque assay described above was performed. All plates were incubated overnight at 37°C. After incubation, PFUs on each plate were enumerated. EOP was calculated as the ratio of the mean PFU on the target bacterium to the mean PFU on the reference host bacterium. Infection efficiency was categorized as follows (35): (i) high efficiency, EOP ≥ 0.5; (ii) medium efficiency, 0.1 ≤ EOP < 0.5; (iii) low efficiency, 0.001 < EOP < 0.1; and (iv) extremely low efficiency, EOP ≤ 0.001.

### Antibiotic susceptibility and growth curve of *K. pneumoniae*

Antibiotic susceptibility of clinical *K. pneumoniae* isolates was determined using the VITEK 2 Compact system with GN67 cards (bioMérieux, France). *K. pneumoniae* isolates were cultured on TSA agar at 35°C for 18–24 h. A bacterial suspension was prepared in 3 mL of 0.45% sterile saline and adjusted to 0.50–0.63 McFarland using a DensiCHEK Plus turbidity meter. Subsequently, 145 μL of the standardized suspension was diluted in 3 mL sterile saline and loaded into AST-GN susceptibility cards. The cards were automatically filled, sealed, and incubated at 35°C for 6–8 h. Bacterial growth in each antimicrobial-containing well was continuously monitored by optical turbidity measurements. Minimum inhibitory concentration (MIC) values for each antimicrobial agent were automatically determined by the system, and susceptibility categories (susceptible, intermediate, or resistant) were interpreted according to the standards of the Clinical and Laboratory Standards Institute (CLSI) (36). Any questionable or unusual results were confirmed using the laboratory’s reference MIC testing method. MICs of antibiotics, including streptomycin, chloramphenicol, kanamycin, ampicillin, tetracycline, amikacin, imipenem, levofloxacin, and ciprofloxacin, were further determined. Antibiotic stock solutions (50 mg/mL) were sterilized by filtration (0.22 μm) and stored at −20°C. *K. pneumoniae* XU1 cultures were grown to OD_600_ ≈ 1.0 and diluted into 96-well plates. Antibiotics were added to achieve an initial concentration of 256 μg/mL, followed by 2-fold serial dilutions. Plates were incubated at 37°C overnight, and OD_600_ was measured to determine bacterial growth. The MIC was defined as the lowest antibiotic concentration with no visible growth. For growth curve analysis, overnight cultures were diluted (1:250) into fresh LB medium and incubated at 37°C with shaking (220 rpm). OD_600_ was measured every 1.5 h for 36 h. All experiments were performed in triplicate. The curve fitting was performed using GraphPad Prism version 10.6.1.

### Induction of persister cells

Persister cells were induced following previously reported methods with minor modifications (37, 38). In this experiment, 20 μL of *K. pneumoniae* XU1 strain frozen at −80°C in glycerol was added to 5 mL of LB medium and incubated at 37°C with agitation (220 rpm) for 12 h to prepare overnight cultures. A fresh 400 μL of XU1 culture was inoculated into 100 mL of LB medium at 37°C for expansion, and OD_600_ was adjusted to 0.2. According to the MIC value of XU1, amikacin was added at concentrations of 0.5×, 1×, 2×, 4×, 6×, 8×, and 10× MIC, while untreated cultures served as controls. Samples were collected at 0, 2, 4, 6, 8, and 10 h for OD_600_ measurement and CFU enumeration. The collected 0.5 mL of the culture solution was centrifuged (8,000 × *g*, 4°C, 3 min) to collect cells, then washed twice with LB liquid (8,000 × *g*, 4°C, 3 min) to remove residual antibiotics, and resuspended in 0.5 mL of LB liquid. A total of 200 μL was transferred to a 96-well plate for 10-fold serial dilution. Finally, 10 mL of the culture solution was pipetted onto LB solid agar plates and incubated overnight at 37°C in a constant temperature incubator. The ratio of the number of surviving bacteria to the number of active bacteria under antibiotic treatment was the induction rate of persister cells.

### Verification of persister phenotype

As proposed by Balaban et al. (39), persister cells were distinguished from resistant mutants based on two criteria: (i) the presence of a biphasic killing curve and (ii) unchanged antibiotic susceptibility after regrowth in antibiotic-free conditions. Therefore, to verify that the cells formed under 10× MIC amikacin exposure were persistent rather than drug-resistant strains, an exposure-time-dependent study was conducted, including both primary and secondary exposures. Based on the induction experiment, individual colonies from the control group and 10× MIC group at 4 h were re-cultured until OD_600_ = 1 and then exposed to 10× MIC amikacin (secondary exposure) or left untreated. Samples were collected again at 0, 2, 4, and 6 h for OD_600_ measurement and counting. If no resistance was observed, the original cells and reactivated cells should exhibit comparable biphasic kill patterns. Additionally, based on the results of the control group and 4× MIC live bacterial count at 4 h post-induction, the live bacterial counts in each group were diluted to a uniform level. Subsequently, the groups were exposed to 4× MIC amikacin (secondary exposure) or left untreated, and samples were collected again at 0, 2, 4, and 6 h for OD_600_ measurement and counting.

Phage validation experiments were performed under standardized initial conditions (OD_600_ = 0.2 and equal CFU counts). The initial conditions were identical to those described above. Four groups were established: control, control + phage, amikacin-treated, and amikacin + phage. At 0, 2, 4, and 6 h, 1 mL of each sample was collected for OD_600_ measurement. A 200 μL mixture was centrifuged at 8,000 × *g* for 3 min, with the supernatant used for phage titration and the bacterial suspension used for live bacterial count determination.

### Cellular ATP measurement

Intracellular ATP levels of *K. pneumoniae* XU1 and persister cells were quantified using an ATP assay kit (Servicebio, G4309-48T) according to the manufacturer’s instructions. Cells were prepared at OD_600_ values of 0.5, 1.0, and 1.5 prior to measurement. All experiments were conducted in triplicate, including control (XU1) and experimental groups (persister cells). The control group received no treatment.

### Optical microscope

Morphological differences between actively growing cells and persister cells were observed using optical microscopy. *K. pneumoniae* XU1 cultures were grown to OD_600_ ≈ 1.0, and persister cells were induced by treatment with 10× MIC amikacin. Ten microliters of bacterial culture was vertically pipetted onto a sterile slide, gently covered with a sterile cover slip to prevent bubble formation, and observed under a 100× oil immersion objective lens mounted on an inverted microscope. The slide position was kept stationary for bacterial observation and imaging. The growth status and division time of bacteria were observed and recorded, with images of the active bacteria and persister cells collected at 10- and 30-min intervals, respectively. The temperature was maintained at 37°C during the experiment to ensure normal bacterial growth.

### Preparation of phage cocktail and *in vitro* killing assay

Three phage strains, vB_KpnM_NB (1–3), were diluted to 1 × 10^9^ PFU/mL and mixed in equal volumes to prepare a cocktail, which was stored at 4°C for future use. The composition ratio of the phage cocktail was derived from previous studies (40). To evaluate the lytic activity of individual phages and the phage cocktail, exponential-phase *K. pneumoniae* XU1 cells and amikacin-induced persister cells were adjusted to the same initial bacterial density (1 × 10^8^ CFU/mL) and infected with individual phages or the phage cocktail at MOIs of 0.1, 1, and 10. Untreated cultures served as controls. Bacterial growth was monitored by measuring optical density (OD_600_) at 2-h intervals over a 24-h period.

### Biofilm inhibition and eradication assays

A colorimetric crystal violet assay was used to quantitatively evaluate the inhibitory effect of the phage cocktail on *K. pneumoniae* biofilm formation, with minor modifications based on previous methods (41). Overnight cultures of *K. pneumoniae* XU1 were prepared. One group was grown to OD_600_ ≈ 1.0 (planktonic cells), while another group was treated with antibiotics for 4 h to induce persister cells. Both groups were diluted 1:200 prior to use. Based on the optimal MOI determined at phage cocktail *in vitro* killing assay previously, the following experimental groups were established in 96-well plates (three replicates per group): (i) control (LB), (ii) antibiotic (10× MIC), (iii) phage cocktail (total MOI = 10), (iv) individual phage (vB_KpnM_NB2, MOI = 10) + antibiotic, and (v) phage cocktail + antibiotic. The same grouping was applied to both planktonic and persister cells. Plates were incubated at 37°C for 12, 24, 36, and 48 h. After incubation, planktonic cells were removed, and wells were gently washed three times with PBS. Biofilms were fixed with 4% methanol for 10 min and air-dried. Subsequently, 200 μL of 0.1% crystal violet was added to each well and stained for 15 min at room temperature. Excess stain was removed by washing with distilled water (5–8 times) until clear. Absorbance was measured at 570 nm using a microplate reader. For biofilm eradication, 200 μL of planktonic or persister XU1 cells was first inoculated into each well of a 96-well plate and incubated statically at 37°C for 24 h to allow biofilm formation. After incubation, planktonic cells were removed, and wells were washed twice with PBS. The same treatment groups as described above were then applied. Subsequent procedures, including incubation, staining, and absorbance measurement, were performed as described above.

### Confocal laser scanning microscope (CLSM)

Biofilm structure and viability were further analyzed using CLSM as previously described (42). Briefly, planktonic or persister XU1 cells (1 × 10^8^ CFU/mL) were inoculated onto confocal glass-bottom dishes and incubated in LB medium at 37°C for 12, 24, 36, and 48 h, with or without phage cocktail and amikacin treatment. Following incubation, biofilm-containing samples were observed using a CLSM system (Leica) with LAS X (v4.7.0) software. Multichannel fluorescence imaging was performed, where green fluorescence was used to visualize the biofilm matrix/background, and blue fluorescence was used to indicate viable bacterial cells.

### Quantitative determination of CPS

We employed the sulfate-phenol method (Greisse G0593F Kit) to determine the capsular polysaccharide (CPS) content in biofilms of non-induced cells and persister cells. *K. pneumoniae* overnight cultures were transferred into LB medium at a 1:250 dilution and cultured to an OD_600_ of approximately 1.0. The retention group was treated with 10× MIC amikacin for 4 h, followed by centrifugation and resuspension to match the OD_600_ of the non-induced group. Both groups were diluted 1:100 and inoculated into a 12-well plate. Biofilms and bacterial cells were collected at 24 and 36 h, centrifuged at 12,000 rpm for 2 min, and washed with PBS. The pellets were resuspended in 2 mL distilled water, boiled at 95–100°C for 2 h, and centrifuged. The supernatant was collected, and 0.2 mL was mixed with 1 mL absolute ethanol, left at 4°C for 1 h to precipitate polysaccharides, and then washed twice with 80% ethanol. The pellet was dissolved in 2 mL distilled water and boiled to obtain the CPS sample solution. Polysaccharide content was determined using the kit instructions. In an EP tube, 200 μL of sample, 100 μL of Reagent I, and 500 μL of concentrated sulfuric acid were added. The blank tube contained distilled water instead of the sample. After a 20-min incubation at 95°C, absorbance was measured at 488 nm. The CPS content was calculated using the standard curve (*y* = 6.24*x* − 0.0327) and expressed as mg/mL.

### Statistical analysis

All experiments were performed in triplicate. Data were analyzed using GraphPad Prism v10.6.1 (GraphPad Software, La Jolla, CA, USA). Statistical significance was assessed using one-way or two-way analysis of variance (ANOVA), followed by Tukey’s multiple comparison test. Results are presented as mean ± standard deviation (SD), and *P* < 0.05 was considered statistically significant.

## RESULTS

### Morphological characterization of phage vB_KpnM_NB

**Fig 1:**
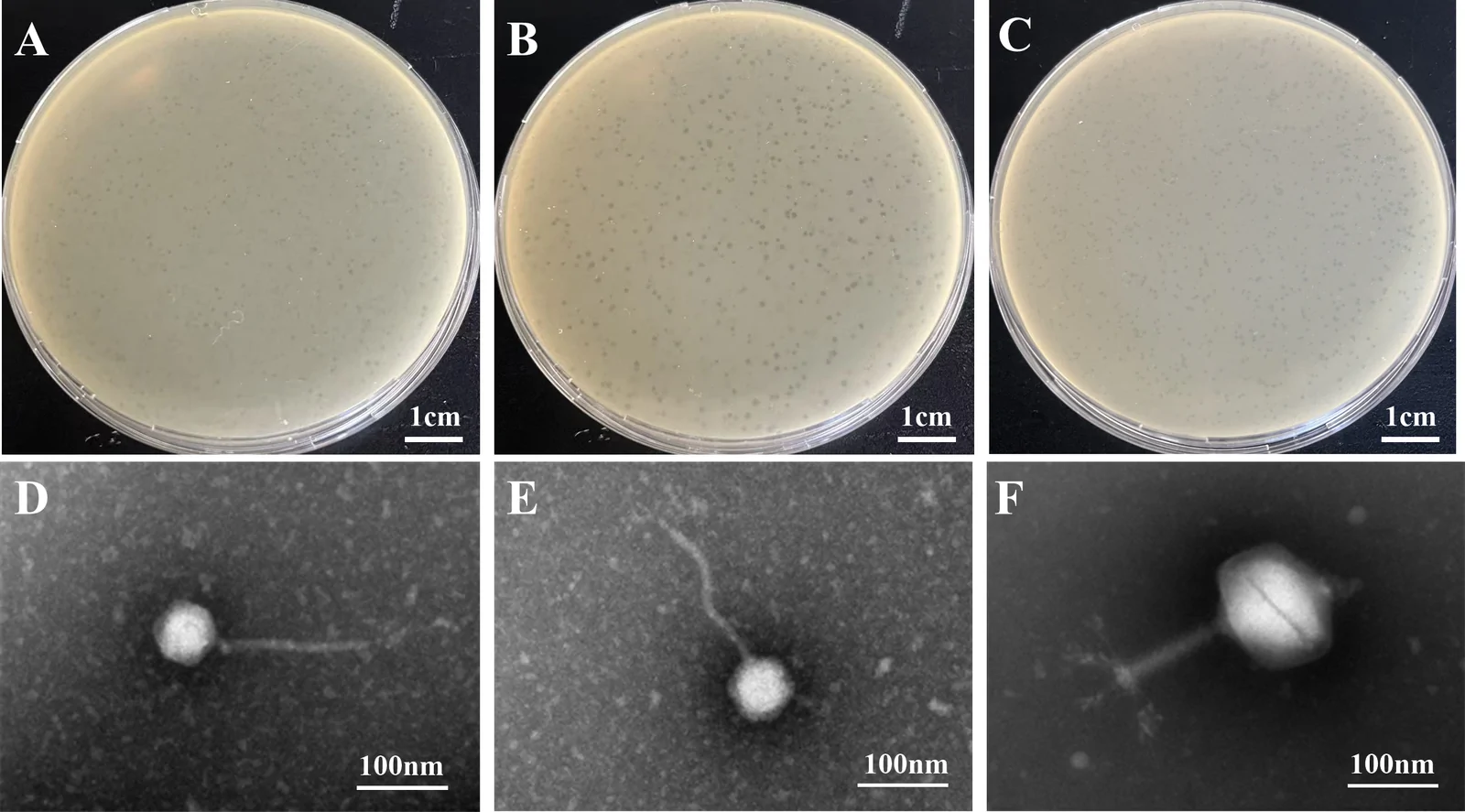
Morphological characterization of phage vB_KpnM_NB. The plaque morphology formed by phages vB_KpnM_NB1 (**A**), vB_KpnM_NB2 (**B**), and vB_KpnM_NB3 (**C**) presents as completely transparent lysed areas, with a scale of 1 cm. TEM images of phages vB_KpnM_NB1 (**D**), vB_KpnM_NB2 (**E**), and vB_KpnM_NB3 (**F**) are displayed at a scale of 100 nm.

To isolate raw materials for the experimental study of lytic phages, samples were collected from river water and domestic wastewater in Ningbo City, Zhejiang Province, China. Using *K. pneumoniae* as the host, three distinct phages were successfully isolated and named vB_KpnM_NB (1–3). These phages were then identified through plaque assays. The results showed that vB_KpnM_NB1 formed clear plaques with diameters of 0.5–1 mm (Fig. 1A), vB_KpnM_NB2 produced plaques ranging from 1 to 2 mm (Fig. 1B), and vB_KpnM_NB3 formed plaques of approximately 1 mm (Fig. 1C). To examine the morphology of the phages, TEM was performed, revealing that all three exhibited typical icosahedral head structures with non-constrictive tails. Specifically, to characterize vB_KpnM_NB1, a head diameter of 60.45 ± 3.87 nm and a tail length of about 110.23 ± 8.34 nm were observed, with a relatively slender tail (Fig. 1D). To analyze vB_KpnM_NB2, a similar head diameter (59.87 ± 4.12 nm) and a tail length of 112.56 ± 9.21 nm were noted (Fig. 1E). To assess vB_KpnM_NB3, a larger head (79.34 ± 5.24 nm) and a longer tail (124.58 ± 10.12 nm) were found, along with distinct baseplate and tail fiber structures characteristic (Fig. 1F).

### Genomic analysis

**Fig 2:**
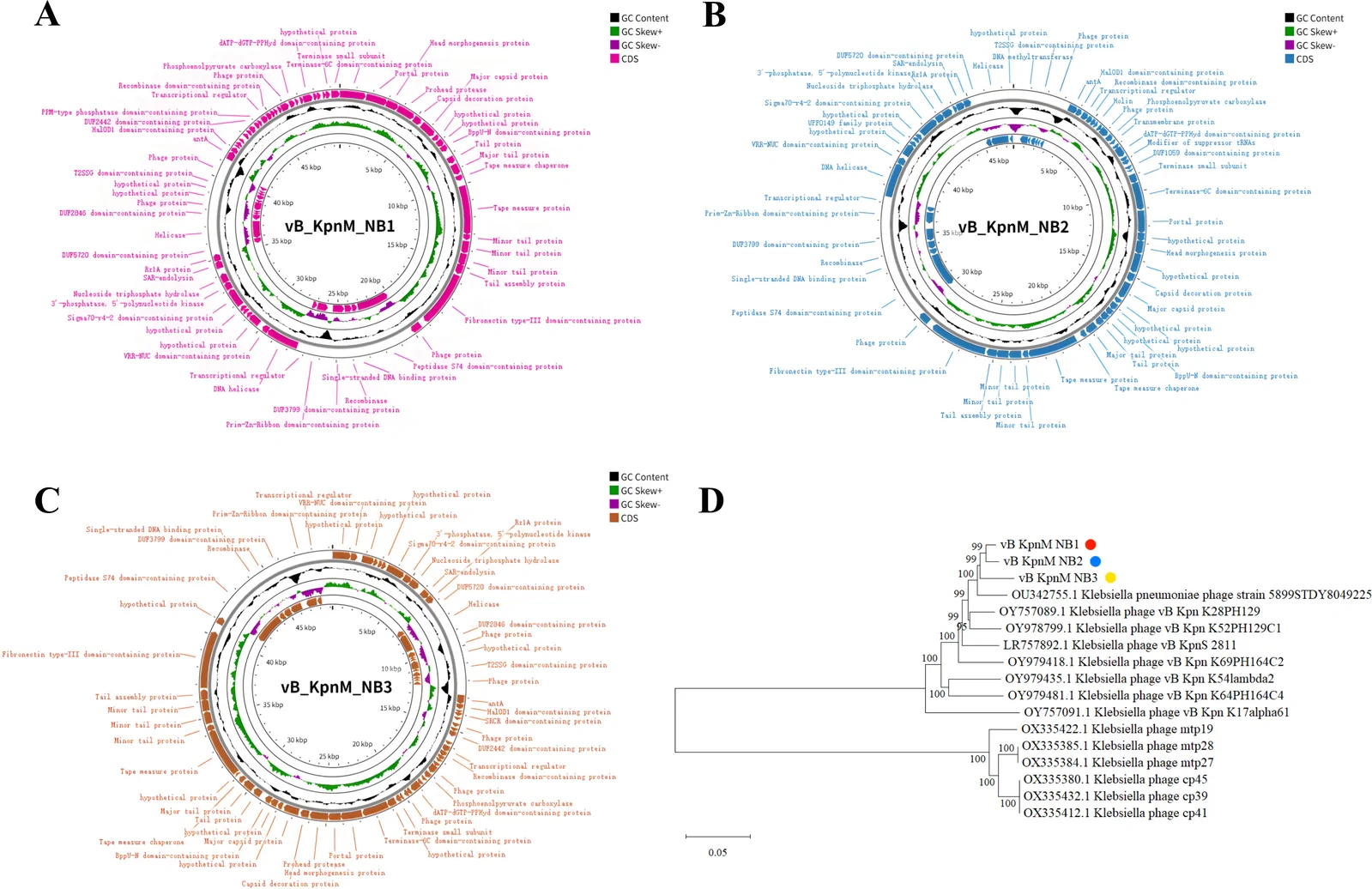
Genomic analysis of phage vB_KpnM_NB. Circular genomic maps and annotation of phages vB_KpnM_NB1 (**A**), vB_KpnM_NB2 (**B**), and vB_KpnM_NB3 (**C**). Each arrow represents an open reading frame (ORF), and the direction of the arrow indicates the transcriptional orientation. From the innermost to the outermost rings: genome scale (inner circle), GC skew (second circle), GC content (third circle), and the genomic positions of coding sequences (CDSs) (outer circle). (**D**) Phylogenetic tree of phage vB_KpnM_NB constructed based on genome alignment using MEGA, with related gene sequences retrieved from the NCBI database. Numbers on the branches represent bootstrap values calculated from 1,000 replicates. Phages vB_KpnM_NB are indicated by circles of different colors.

BLASTn alignment revealed that all three phages we identified exhibited the highest similarity to phage vB_Kpn_GBH029 (GenBank NCBI reference sequence: OU342755.1). Based on morphological and BLAST sequence comparisons, the three phages are likely classified under the class Caudoviricetes, order Caudovirales, and family Drexlerviridae. Whole-genome sequencing (WGS) of the phage genomes revealed significant differences in DNA conformation, genome size, GC content, and the number of predicted open reading frames (ORFs) (Fig. 2A through C), as shown in Table 1. Notably, no tRNA-coding genes were detected in any of the three phages. Genome annotation revealed that these phages do not carry integrase, recombinase, or repressor protein-coding genes, indicating that they are strictly lytic phages. Furthermore, no genes related to antibiotic resistance or virulence factors were found, nor were homologs of lysogenic or virulence transfer-related genes identified. These findings suggest that these phages have potential safety for therapeutic applications. Based on whole-genome sequences, a phylogenetic tree was constructed using the Neighbor-Joining method to clarify the evolutionary classification of the three phages (Fig. 2D). The results showed that vB_KpnM_NB1, vB_KpnM_NB2, and vB_KpnM_NB3 clustered tightly into a single clade, with bootstrap values of 99–100%, indicating extremely close genetic relatedness and that they belong to the same evolutionary lineage. This clade further grouped with multiple previously reported *K. pneumoniae* myophages (e.g., OU342755.1, OY757089.1), forming a highly supported monophyletic group. However, some divergence was still observed within the phylogenetic tree, supporting differences in evolutionary background and potential functional variation among the phages.

**TABLE 1:** Characterization of phage vB_KpnM_NB genomes

Phage name	Conformational form of DNA	Genome length (bp)	GC content (%)	No. of ORFs	No. of hypothetical proteins
vB_KpnM_NB1	Circular dsDNA	49,575	51	76	37
vB_KpnM_NB2	Linear dsDNA	47,675	52	72	33
vB_KpnM_NB3	Circular dsDNA	49,225	49	76	37

### Biological characteristics of phage vB_KpnM_NB

**Fig 3:**
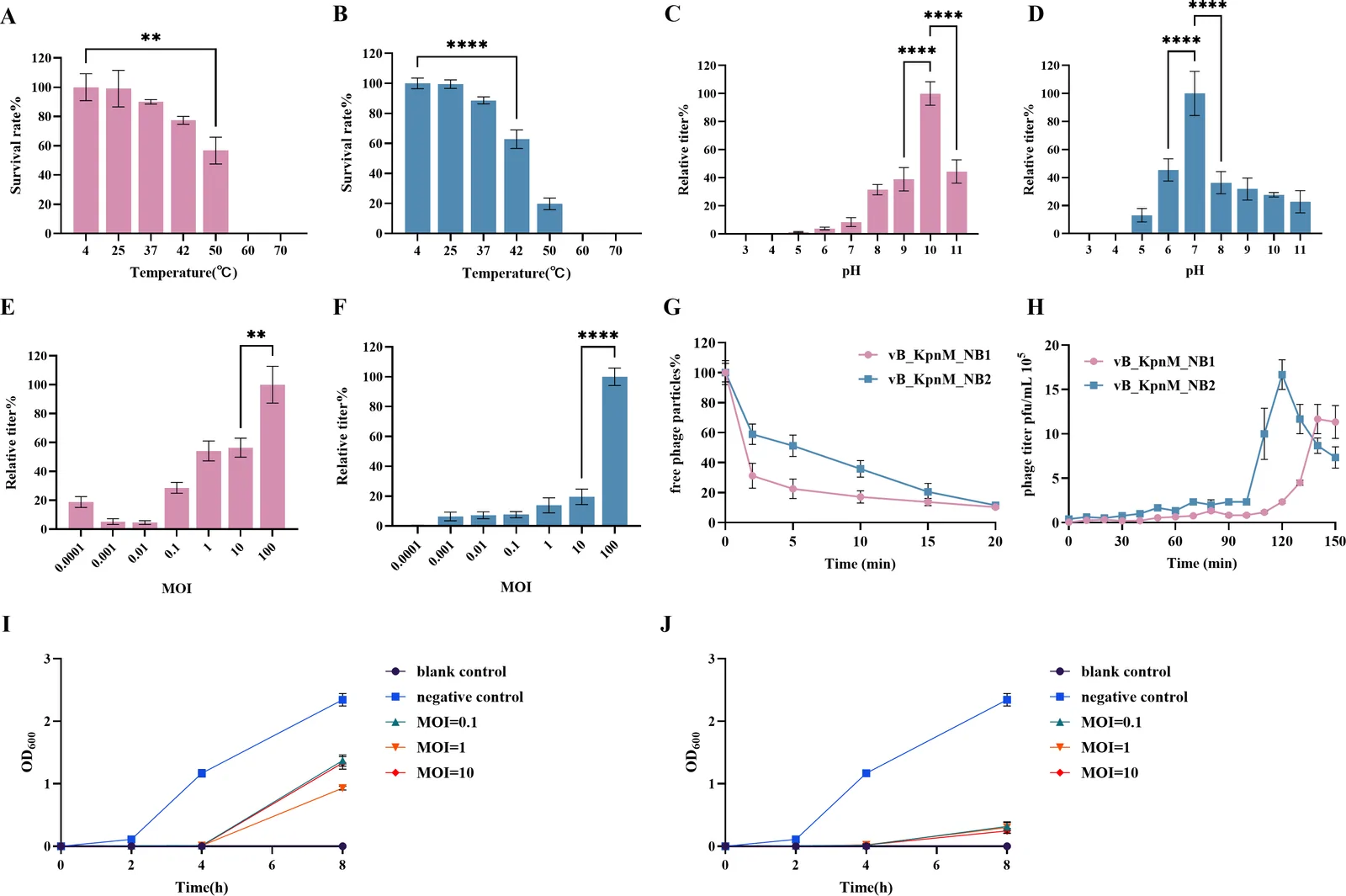
Biological characteristics of phages vB_KpnM_NB (1 and 2). Thermal stability of phages vB_KpnM_NB1 (**A**) and vB_KpnM_NB2 (**B**) after incubation at different temperatures (4–70°C) for 1 h. The pH stability of phages vB_KpnM_NB1 (**C**) and vB_KpnM_NB2 (**D**) under different pH conditions (pH 3–11). The MOI optimization of vB_KpnM_NB1 (**E**) and vB_KpnM_NB2 (**F**). (**G**) Adsorption curves of phages vB_KpnM_NB (1 and 2) on host bacteria. (**H**) One-step growth curves of phages vB_KpnM_NB (1 and 2). Antimicrobial activity of vB_KpnM_NB1 (**I**) and vB_KpnM_NB2 (**J**) at different MOIs, with bacterial growth levels reflected by OD_600_ values. Data are presented as mean ± SD of three independent experiments. Statistical analyses were performed using one-way ANOVA with Dunnett’s post hoc test. *P < 0.05, **P < 0.01, ***P < 0.001, and ****P < 0.0001.

The environmental stability and replication characteristics of phages are key indicators determining their antibacterial potential (43). Therefore, systematic evaluations were conducted on temperature, pH, adsorption, and one-step growth curves. To evaluate the stability of phages vB_KpnM_NB1 and vB_KpnM_NB2 under different environmental conditions, phage suspensions were incubated at various temperatures and pH values, followed by determination of phage titers. The results showed that both phages remained stable within the temperature range of 4°C–37°C, with no significant change in titer. However, a marked decline in phage activity was observed at 50°C. Phage vB_KpnM_NB2 was completely inactivated after incubation at 60°C for 1 h (Fig. 3B), whereas vB_KpnM_NB1 retained 1.73% of its initial titer under the same conditions and was completely inactivated at 70°C (Fig. 3A), indicating moderate thermal sensitivity at elevated temperatures. Regarding pH stability, vB_KpnM_NB1 exhibited optimal activity at pH 10 and was completely inactivated at pH 3 and 12 (Fig. 3C). In contrast, vB_KpnM_NB2 showed the highest titer at neutral pH (pH 7) and lost activity entirely under acidic conditions (pH 3) (Fig. 3D). Overall, vB_KpnM_NB1 displayed better tolerance to alkaline environments but was highly sensitive to acidic conditions, whereas vB_KpnM_NB2 was more stable under neutral conditions. To investigate the MOI, phages were mixed with host bacteria at varying ratios. Phage vB_KpnM_NB1 demonstrated the highest infection efficiency at an MOI of 100. As the MOI was increased from 0.0001 to 100, the phage yield initially declined before increasing, reaching a minimum at an MOI of 0.01 (Fig. 3E). For vB_KpnM_NB2, the optimal MOI was also found to be 100, with phage production showing a positive correlation with the MOI (Fig. 3F).

For adsorption characteristics, the results demonstrated that after incubation at 25°C for 20 min, 89.69% of vB_KpnM_NB1 and 88.46% of vB_KpnM_NB2 particles were adsorbed onto host cells, indicating a time-dependent adsorption process (Fig. 3G). To assess the replication dynamics, a one-step growth curve analysis was performed, showing that vB_KpnM_NB1 had a longer latent period (110 min) and a larger burst size (196.88 ± 41.83 PFU/cell) compared to vB_KpnM_NB2, which exhibited a latent period of 100 min and a burst size of 35.83 ± 10.36 PFU/cell (Fig. 3H). These findings suggest distinct replication profiles between the two phages.

### Phages’ lytic capacity *in vitro*

Both vB_KpnM_NB1 and vB_KpnM_NB2 significantly inhibited the growth of the target strain, with suppression lasting for approximately 4 h (Fig. 3I and J). Specifically, at an MOI of 0.1, vB_KpnM_NB1 reduced bacterial growth by 41.47% ± 7.60%, while vB_KpnM_NB2 demonstrated a more pronounced inhibition, reducing bacterial growth by 86.44% ± 5.11%. Among the two, vB_KpnM_NB2 exhibited superior antibacterial activity. Both phages effectively inhibited bacterial growth even at a low MOI of 0.1, suggesting their potential for use in phage therapy.

### Determination of phage host range

The role of phages in the treatment of *K. pneumoniae* infections, particularly their host range, has become an increasingly studied area. While previous research has primarily focused on the lytic activity of individual phages, understanding their broad-spectrum activity across different bacterial strains is crucial for practical applications (44). To address this, we investigated the host range of six *Klebsiella*-specific phages—vB_KpnM_NB1, vB_KpnM_NB2, vB_KpnM_NB3, ZRP-2, ZRP-3, and Sg-1P—by testing their lytic activity against a diverse range of bacterial strains. These strains included nine *K. pneumoniae* isolates as well as other bacterial species such as *E. coli*, *E. cloacae*, *P. aeruginosa*, and *V. parahaemolyticus* (Table 2). As expected, all six phages exhibited a high degree of specificity for *K. pneumoniae*, showing lytic activity exclusively against *K. pneumoniae* strains ZR and XU1, with no lysis observed in the other bacterial species tested. These findings indicate that the isolated phages are primarily targeted at *Klebsiella* species, minimizing the risk of off-target effects on commensal microbiota or non-target pathogens. After preliminary spot assay screening, host strains that formed plaques were further evaluated for their EOP. The infectivity of five phages against three clinical *K. pneumoniae* isolates was assessed (Table 3). Phage vB_KpnM_NB3 exhibited the broadest host range, showing highly efficient infection of strains XU1 (EOP = 1.0) and ZR (EOP = 0.95), and moderate infectivity against strain Sg-1 (EOP = 0.55). In contrast, vB_KpnM_NB1 and vB_KpnM_NB2 showed markedly reduced infectivity toward ZR (EOP = 0.32 and 0.11, respectively) and no detectable activity against Sg-1 (EOP < 10^−4^). These results indicate that the tested phages display clear host specificity, suggesting that differences in recognition of strain-specific surface components (such as capsular polysaccharides or outer membrane receptors) may underlie their distinct infection spectra.

**TABLE 2:** Efficiency of lysis by phages against 18 strains of clinical *K. pneumoniae^a^*

Species	Strain name	PFU/mL					
		vB_KpnM_NB1	vB_KpnM_NB2	vB_KpnM_NB3	ZRP-2	ZRP-3	Sg-1P
*K. pneumoniae*	448	–	–	–	–	–	–
*K. pneumoniae*	449	–	–	–	–	–	–
*K. pneumoniae*	456	–	–	–	–	–	–
*K. pneumoniae*	ZR	++ 3.5 × 10^7^	++ 1.5 × 10^7^	+ 5.2 × 10^6^	++ 2.1 × 10^8^	++ 1.5 × 10^8^	++ 1.8 × 10^7^
*K. pneumoniae*	XU1	++ 1.1 × 10^8^	++ 1.4 × 10^8^	+ 5.5 × 10^6^	++ 1.1 × 10^8^	+ 2.8 × 10^7^	++ 1.8 × 10^7^
*K. pneumoniae*	Sg-1	–	–	+ 3.0 × 10^6^	–	–	–
*K. pneumoniae*	PNE	–	–	–	–	–	–
*K. pneumoniae*	LU1785	–	–	–	–	–	–
*K. pneumoniae*	LU1787	–	–	–	–	–	–
*E. coli*	IC 82	–	–	–	–	–	–
*E. coli*	IC 83	–	–	–	–	–	–
*E. coli*	IC 85	–	–	–	–	–	–
*E. coli*	IC 97	–	–	–	–	–	–
*E. coli*	NDY	–	–	–	–	–	–
*E. coli*	Y6	–	–	–	–	–	–
*E. cloacae*	LC1	–	–	–	–	–	–
*P. aeruginosa*	PA1	–	–	–	–	–	–
*V. parahaemolyticus*	RIMD	–	–	–	–	–	–

^*a*^ ++ indicates the formation of clear plaques, + indicates the formation of turbid plaques, and – indicates that the bacterial strain is insensitive to phage activity. Identical results were obtained in three independent replicates. The killing efficiency of each phage strain was determined by the EOP assay, and PFU values presented in the table represent the mean of three replicate experiments. Grey shading marks the strains susceptible to lysis by the three phages investigated in this study.

**TABLE 3:** Calculation results for efficiency of plating (EOP)^*a*^

Species	Strain name	vB_KpnM_NB1	vB_KpnM_NB2	vB_KpnM_NB3	ZRP-2	ZRP-3
*K. pneumoniae*	ZR	0.32	0.11	0.95	1.0	1.0
*K. pneumoniae*	XU1	1.0	1.0	1.0	0.52	0.19
*K. pneumoniae*	Sg-1	–	–	0.55	–	–

^*a*^ The EOP value is defined as the ratio of the PFU/mL of the test bacterium to that of the host bacterium. – indicates EOP < 0.0001.

### Antibiotic susceptibility and growth curve of *K. pneumoniae*

**Fig 4:**
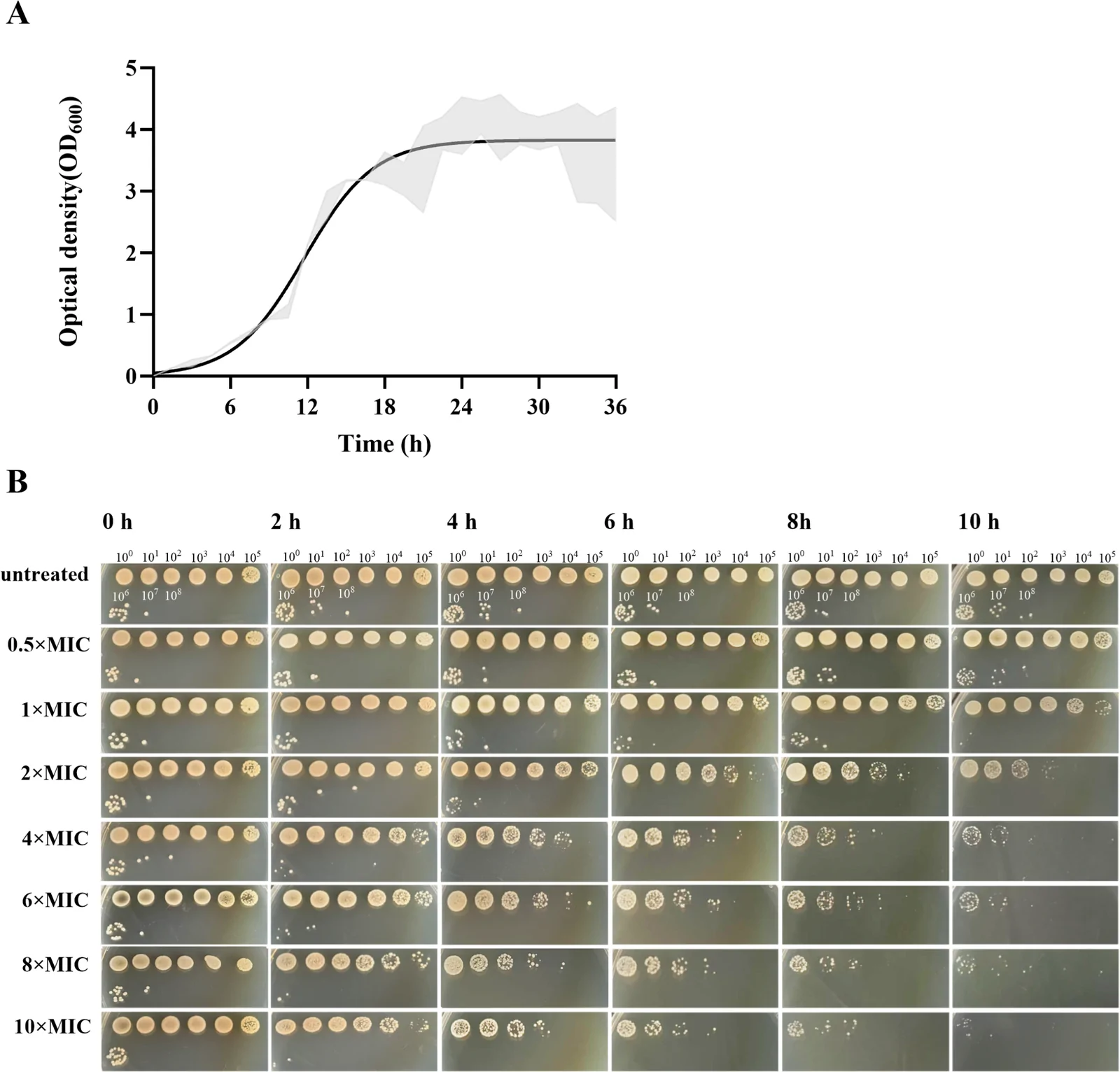
Formation of amikacin-induced persister cells in *K. pneumoniae*. (**A**) Growth curve of *K. pneumoniae*. Data are presented as mean ± SD (*n* = 3). (**B**) Exponentially growing *K. pneumoniae* XU1 cells were exposed to amikacin at 0.5×, 1×, 2×, 4×, 6×, 8×, and 10× MIC for 0, 2, 4, 6, 8, and 10 h. Serial dilutions ranging from the original sample to 10^8^-fold dilution were used. The results showed that no significant difference in viable counts was observed at 0 h. From 4 h onward, viable cells decreased in a concentration- and time-dependent manner, with significant differences among groups. After 10 h at 10× MIC, 16 CFU/mL surviving cells remained, suggesting persister cells induction and enrichment.

The increasing prevalence of antibiotic-resistant *K. pneumoniae* strains underscores the need for alternative therapeutic strategies to combat persistent infections. A key factor in the persistence of bacterial infections is the ability of certain cells to enter a dormant state, known as persister cells, which are highly tolerant to antibiotic treatment (45). To better understand the antibiotic resistance characteristics of *K. pneumoniae*, we assessed the MICs of 23 commonly used antibiotics against a clinical *K. pneumoniae* isolate using both the VITEK 2 Compact system and laboratory-based MIC assays. The results are summarized in Table 4. Based on standard antimicrobial susceptibility guidelines, the isolate was found to be resistant to 22 of the tested antibiotics, with intermediate susceptibility observed only to amikacin, an aminoglycoside antibiotic. This selective resistance pattern suggested that amikacin might be an ideal candidate for inducing persister cell formation. Consequently, amikacin was selected for subsequent experiments focused on persister cell induction, particularly in experiments involving actively growing cells. To determine the bacterial growth phases, the growth curve of *K. pneumoniae* strain XU1 was monitored (Fig. 4A). At 37°C, the strain entered the exponential (logarithmic) growth phase at approximately 10.5 h, which was selected as the baseline time point for subsequent experiments requiring actively growing cells.

**TABLE 4:** Antibiotic susceptibility profile of the clinical *K. pneumoniae* isolate against commonly used antimicrobial agents

Antibiotic	Initialism	Drug susceptibility test criteria (mg/mL)		MIC	Result determination
		S (sensitive)	R (resistant)		
Amikacin	AMK	16	64	4	I (intermediate)
Ampicillin	‌AMP	8	32	≥32	R
Aztreonam	ATM	4	16	16	R
Bactrim	CO SMZ	2/38	4/76	≥320	R
Cefazolin	CFZ	2	8	≥64	R
Cefepime	FEP	2	16	32	R
Cefotetan	CTT	16	64	≥64	R
Ceftazidime	CAZ	4	16	≥64	R
Ceftriaxone	CR	1	4	≥64	R
Ciprofloxacin	CIP	0.25	1	≥4	R
Ertapenem	ETP	0.5	2	≥8	R
Furadantin	F	–	–	256	R
Gentamicin	CN	4	16	≥16	R
Imipenem	IPM	1	4	2	R
Levofloxacin	LVX	0.5	2	≥8	R
Piperacillin	PIP	8	32	32	R
Streptomycin	STR	6	16	4,096	R
Chloramphenicol	CHL	8	32	256	R
Kanamycin	KAN	16	64	>2,048	R
Ampicillin	AM	8	32	>2,048	R
Achromycin	TET	4	16	1,024	R

### Induction of persister cells

In time-dependent amikacin killing assays, dormant persister cells were formed within the population of the *K. pneumoniae*. Specifically, bacterial cultures were exposed to increasing concentrations of amikacin (0.5×, 1×, 2×, 4×, 6×, 8×, and 10× MIC) for 0–10 h. The number of viable cells decreased progressively with increasing antibiotic concentration and prolonged exposure time. Notably, after treatment with 10× MIC amikacin for 10 h, a small subpopulation of cells (16 CFU/mL) remained viable, despite the substantial reduction in the overall bacterial population. This residual survival strongly suggests the induction and enrichment of persister cells rather than complete eradication (Fig. 4B).

### Verification of persister cells

**Fig 5:**
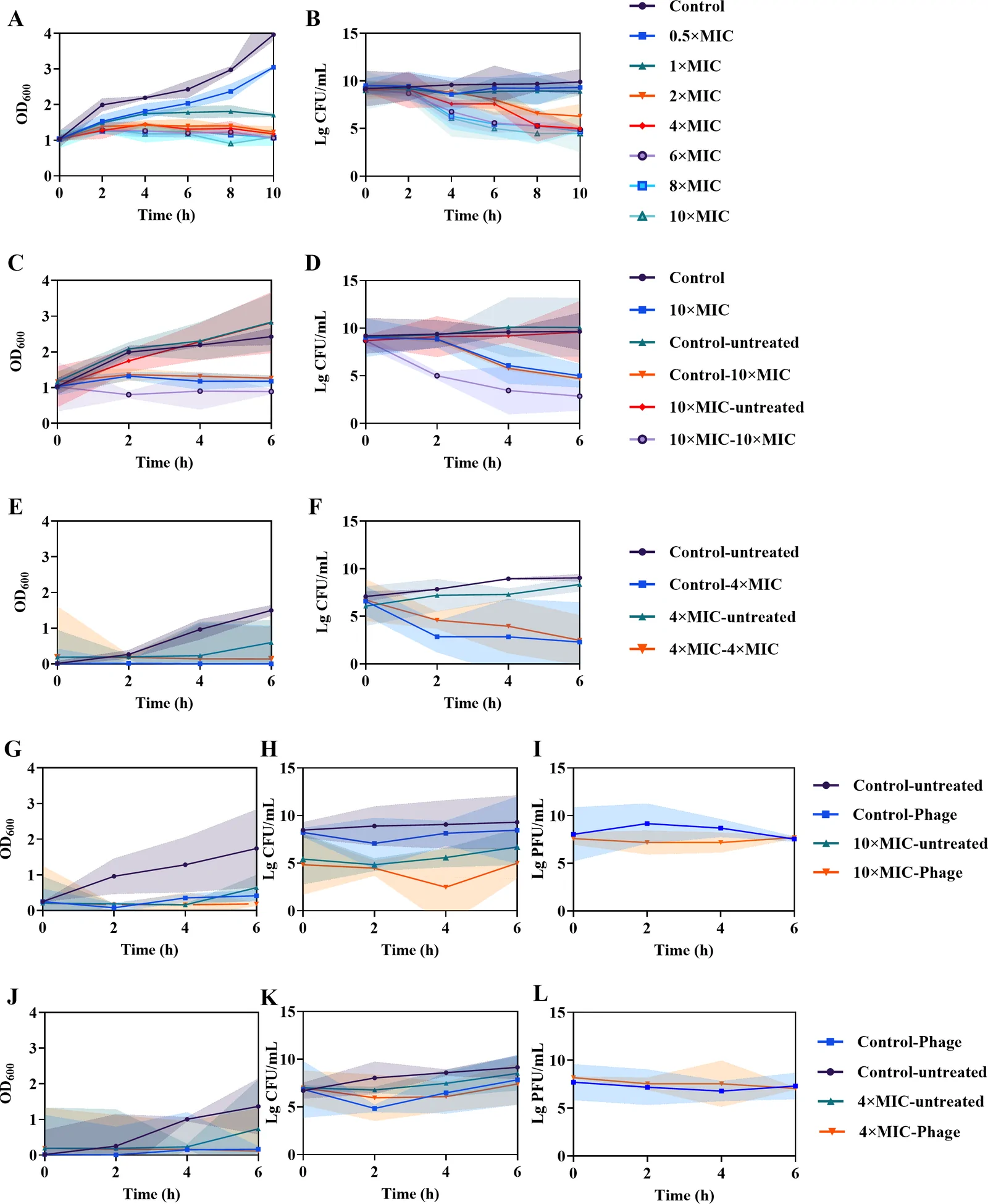
Verification of the effects of amikacin and phage on persister cells. The experiments included primary exposure and secondary exposure assays. During primary exposure, only the effect of amikacin on persister cells was evaluated by measuring OD_600_ (**A**) and bacterial counts (**B**). For secondary exposure to amikacin, two initial conditions were examined: equal initial OD_600_ (OD_600_ = 1), with measurements of OD_600_ (**C**) and bacterial counts (**D**), and equal initial viable cell counts, with measurements of OD_600_ (**E**) and bacterial counts (**F**). For secondary exposure to phages, two initial conditions were also assessed: equal initial OD_600_ (OD_600_ = 0.2), with measurements of OD_600_ (**G**), bacterial counts (**H**), and phage titers (**I**), and equal initial viable cell counts, with measurements of OD_600_ (**J**), bacterial counts (**K**), and phage titers (**L**). Data are presented as mean ± SD (*n* = 3).

Against this background, can amikacin-induced bacterial populations display the hallmarks of typical persister cells and satisfy our ideal persister model? To verify this, we assessed the bactericidal effects of amikacin and phages on persister cells by monitoring OD_600_, CFU counts, and phage titers. We conducted both initial and secondary exposure experiments for multidimensional validation. During the initial exposure (Fig. 5A and B), the control group exhibited continuous increases in bacterial density (OD_600_) and viable cell counts, with OD_600_ rising from 1.032 to 3.960. In contrast, all amikacin-treated groups (0.5–10× MIC) maintained OD_600_ values below 3.050, with the 10× MIC group reaching the lowest OD_600_ of 0.906 at 8 h. As the concentration of amikacin increased, bacterial counts decreased significantly, especially in the 8× MIC and 10× MIC groups, where CFU counts dropped from 9.00 lg CFU/mL to 4.70 lg CFU/mL and 4.48 lg CFU/mL, respectively. This indicates a positive correlation between antibiotic concentration and bactericidal efficacy. Over time, a typical time-dependent killing pattern was observed: after 10× MIC amikacin treatment, viable cell counts rapidly decreased within the first 6 h, followed by a stabilization phase (0 h: 9.00 lg CFU/mL → 6 h: 5.00 lg CFU/mL → 10 h: 4.48 lg CFU/mL).

The second exposure experiment examined two initial conditions (Fig. 5C and D): when the initial OD_600_ was set to 1, the control and untreated groups exhibited bacterial growth similar to that observed in the first exposure, with OD_600_ increasing from 1.168 to 2.832. In contrast, persister cells induced by 10× MIC of amikacin showed rapid recovery, with OD_600_ increasing from 1.020 to 2.820. Upon re-exposure to 10× MIC, both OD_600_ and CFU remained at low levels, with OD_600_ at 0.888 and CFU dropping to 2.85 lg CFU/mL, confirming that amikacin effectively inhibited bacterial proliferation. Under conditions of equal initial viable cell counts (Fig. 5E and F), bacterial counts in the amikacin-treated group continued to decrease, further verifying its bactericidal activity against persister cells. Phage treatment was similarly assessed under both initial conditions: when the initial OD_600_ was equal (0.2) (Fig. 5G through I), the phage-treated groups maintained OD_600_ values consistently below 0.5, while phage titers (lg PFU/mL) remained between 8 and 9, indicating that the phages not only lyse recovering persister cells but also maintain their replicative capacity. Under equal initial viable cell counts (Fig. 5J through L), the bacterial density in the phage-treated persister cell group (0.113) was significantly lower than that in the control group (0.736), and the phage titer did not show a noticeable decline, further confirming the sustained bactericidal effect of phages on persister cells.

In conclusion, both amikacin and phages effectively suppress or eliminate persister cells. Significantly, persister cells exhibited recovery ability, and the regenerated population retained similar antibiotic sensitivity to the original population, confirming that the surviving cells were persisters rather than antibiotic-resistant mutants.

### Intracellular ATP measurement

**Fig 6:**
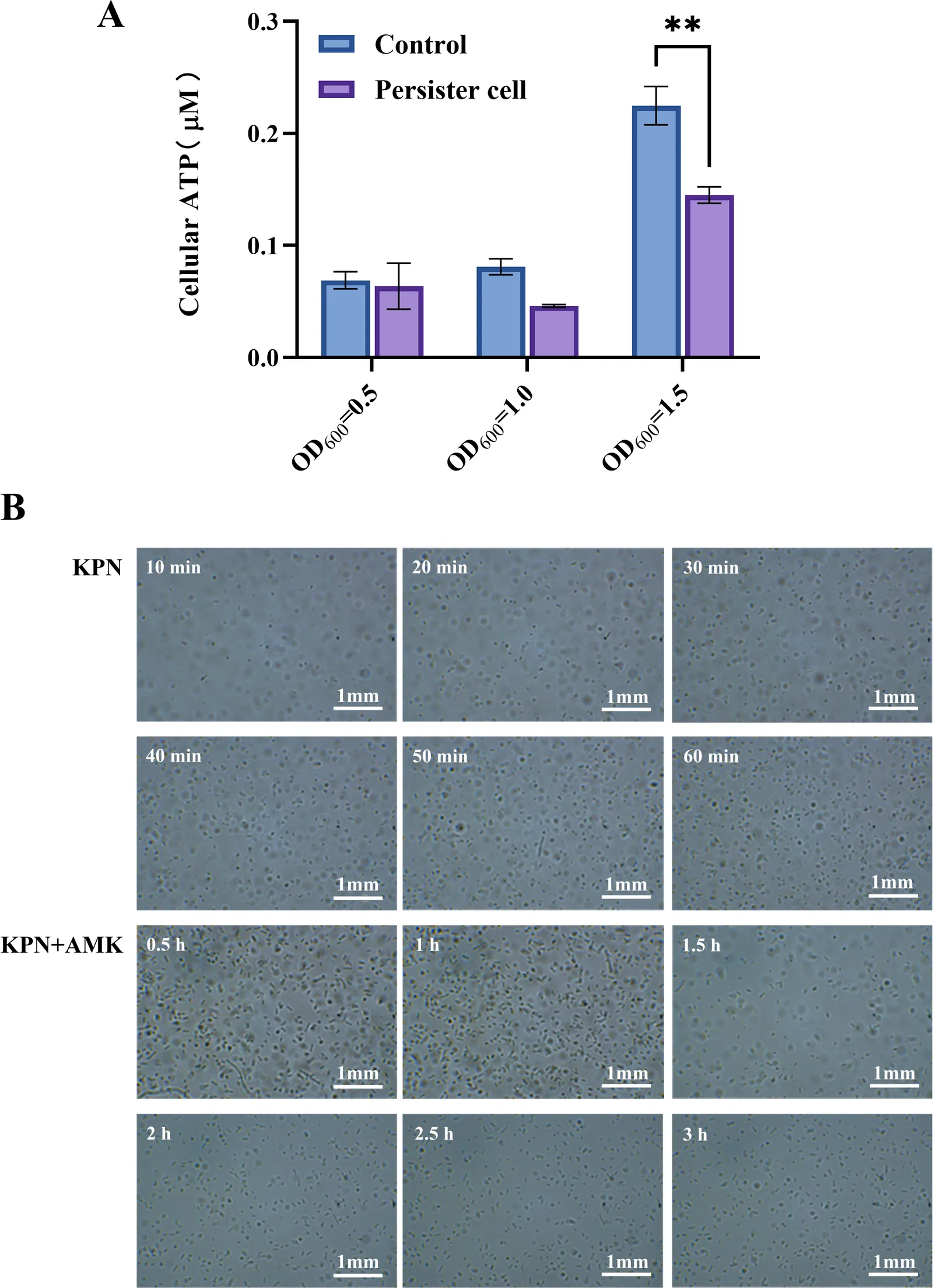
Verification of persister cells. (**A**) Intracellular ATP levels of *K. pneumoniae* and antibiotic-induced persister cells. ATP levels were measured using an ATP assay kit in *K. pneumoniae* XU1 and antibiotic-induced persister cells cultured to OD_600_ values of 1.5, 1.0, and 0.5. Data are presented as mean ± standard deviation (*n* = 3). (**B**) Optical microscopy observations of the growth of active *K. pneumoniae* cells cultured in fresh LB medium and the formation of persister cells induced by 10× MIC amikacin. The acquisition time of each image is indicated in the upper-left corner, and the scale bar is shown in the lower-right corner.

We sought to understand whether these cells possess unique energy characteristics that contribute to their antibiotic tolerance. Previous studies have suggested that persister cells, which are metabolically inactive and highly resistant to antibiotics, may exist in a low-energy state (46). We aimed to determine how the metabolic activity of induced persister cells compares to that of actively growing bacterial populations. To achieve this, we quantified intracellular ATP levels at various growth stages, including data for cells with OD_600_ values of 1.5, 1.0, and 0.5. The results shown in Fig. 6A indicate that ATP levels in antibiotic-induced persister cells were consistently lower than those in *K. pneumoniae* cells that were actively growing prior to induction. Specifically, at an OD_600_ of 1.5, ATP levels in persister cells were reduced by 36.05% compared to actively growing cells. Moreover, as the OD_600_ value decreased, ATP levels progressively declined, demonstrating a clear correlation between cell density and metabolic activity. These findings suggest that persister cells exist in a low-energy metabolic state, which is consistent with their dormant phenotype and contributes to their enhanced tolerance to antibiotic treatment.

### Optical microscopy observation

Observation of active bacteria and persister cell growth using an optical microscope serves as another validation method. For cells in the exponential growth phase, all bacteria exhibited immediate growth upon observation. Cell proliferation was initiated within 20 min, and the cell population increased nearly 10-fold after 60 min. In contrast, in the presence of 10× MIC amikacin, the majority of susceptible cells were eliminated within 1.5 h, leaving approximately 13% of the population surviving. These surviving cells exhibited delayed and slow division, indicating their persistence under antibiotic stress. After the gradual decline in antibiotic pressure, after 3 h, these surviving cells gradually resumed metabolic activity and cell division, transitioning from a dormant state to active proliferation. This observation further supports that the residual subpopulation represents persister cells capable of resuscitation (Fig. 6B).

### *In vitro* bactericidal activity of individual phage and phage cocktail

**Fig 7:**
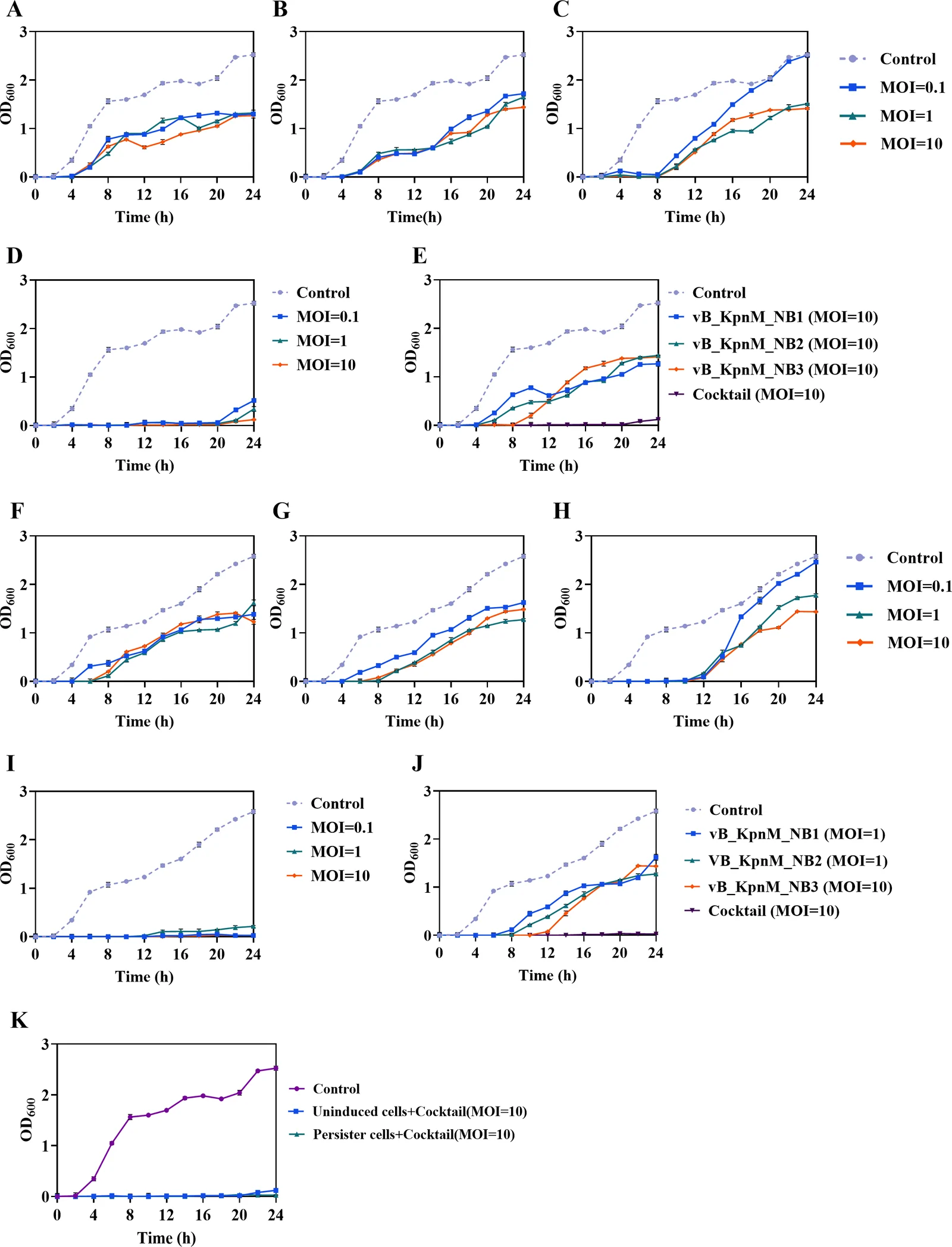
*In vitro* infection kinetics (24 h) of individual phages and phage cocktail against *K. pneumoniae* without antibiotic induction and persister cells. Non-induced *K. pneumoniae* was mixed with individual phages vB_KpnM_NB1 (**A**), vB_KpnM_NB2 (**B**), vB_KpnM_NB3 (**C**), or the phage cocktail (**D**), and optical density (OD_600_) was recorded as an indicator of bacterial growth. Measurements were taken at 2-h intervals over 24 h. (**E**) Comparison of the bactericidal efficacy of individual phages and the phage cocktail at their optimal MOI against non-induced *K. pneumoniae*. Persister cells of *K. pneumoniae* were incubated with phages vB_KpnM_NB1 (**F**), vB_KpnM_NB2 (**G**), vB_KpnM_NB3 (**H**), or the phage cocktail (**I**), and OD_600_ was recorded to monitor bacterial growth. (**J**) Comparison of the bactericidal efficacy of individual phages and the phage cocktail at their optimal MOI against persister cells. (**K**) Comparative bactericidal performance of the phage cocktail against non-persister and persister *K. pneumoniae* cells.

To investigate the bactericidal effects of the phage cocktail, we performed experiments using individual phages and a phage cocktail consisting of three different phages against both normal *K. pneumoniae* cells and persister cells. Overall, phage treatment significantly inhibited bacterial growth, with all phages exhibiting similar inhibitory trends. Importantly, the phage cocktail demonstrated superior bactericidal efficiency compared to individual phages, regardless of whether the cells were in a normal or persister state (Fig. 7A through J). As shown in Fig. 7E, the phage cocktail achieved a killing efficiency of 95.20% ± 4.04% in normal cells, which was significantly higher than that of individual phages vB_KpnM_NB1 (49.76% ± 2.53%), vB_KpnM_NB2 (42.92% ± 0.40%), and vB_KpnM_NB3 (43.97% ± 0.57%). In Fig. 7J, in the persister cell group, the phage cocktail exhibited a killing efficiency of 99.00% ± 1.59%, again outperforming vB_KpnM_NB1 (37.15% ± 2.79%), vB_KpnM_NB2 (50.67% ± 1.71%), and vB_KpnM_NB3 (44.32% ± 1.59%). Figure 7K shows that the phage cocktail exhibited strong bactericidal activity against both planktonic and persister cells, with OD_600_ values significantly reduced to 0.121 and 0.026, respectively. In conclusion, phages demonstrate strong antibacterial activity against both normal and persister cells. Increasing phage concentration or using phage combination therapy can partially enhance the inhibition of persister cells. Compared to individual phage therapy, the phage cocktail shows broader antimicrobial activity and delays the emergence of resistance.

### Biofilm inhibition and eradication assays

Biofilms serve as critical survival structures for bacteria to resist external stress and enhance drug resistance. Their three-dimensional architecture and dense matrix significantly reduce the efficacy of phage and antibiotic interactions (47, 48). To quantify the antibacterial effect of phage cocktails in a biofilm environment, crystal violet staining was used to measure biofilm biomass. *K. pneumoniae* strains in both normal and persister states were treated with a phage cocktail composed of vB_KpnM_NB (1–3) at a 1:1:1 ratio at a MOI of 10, synergizing with antibiotics.

**Fig 8:**
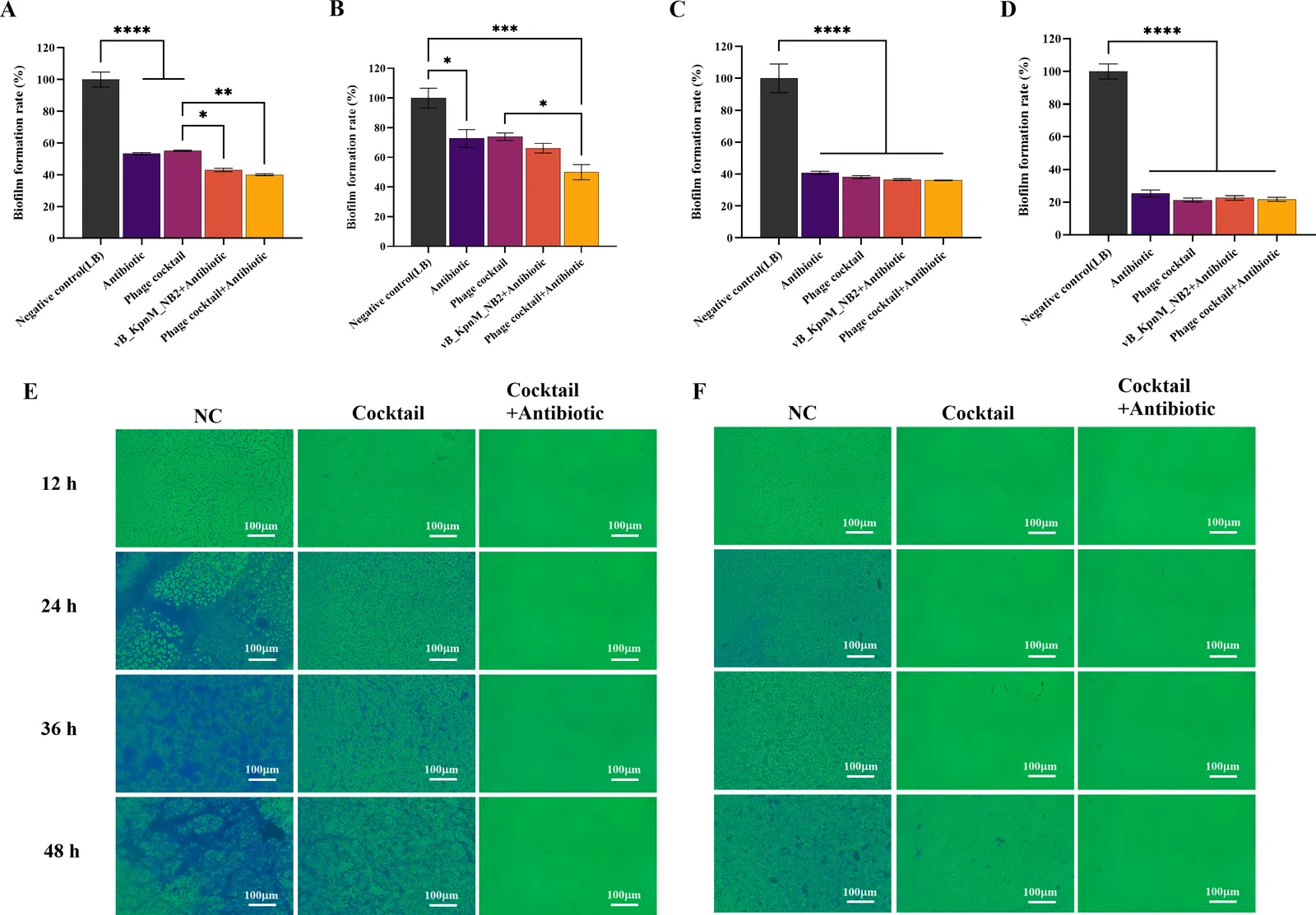
Effects of antibiotics, phage cocktail, and their combination on the biofilms formed by non-induced cells and persister cells. In order to measure inhibition of biofilm formation, non-induced *K. pneumoniae* cells (**A**) and persister cells (**B**) were cultured to form biofilms and co-incubated with antibiotics and phages for 12 h. At the end of the experiment, the biofilms were stained with crystal violet and measured at a wavelength of 570 nm using a spectrophotometer. In order to measure phage killing in mature biofilms, *K. pneumoniae* non-induced cells (**C**) and persister cells (**D**) were cultured for 24 h to establish mature biofilms. Antibiotics and phages were then introduced, and the biofilms were incubated for an additional 12 h. At the end of the experiment, the biofilms were stained with crystal violet and measured at a wavelength of 570 nm. All experimental data were statistically analyzed using two-way ANOVA. *P < 0.05, **P < 0.01, ***P < 0.001, and ****P < 0.0001. Confocal laser scanning microscopy (CLSM) images of *K. pneumoniae* after 12, 24, 36, and 48 h of incubation. Biofilms formed by sensitive cells (**E**) and persister cells (**F**) under negative control conditions, phage cocktail treatment, or combined antibiotic treatment. Green: background; blue: bacterial aggregates/biofilm formation. The scale bar in each image represents 100 μm.

In the biofilm inhibition experiment, the interventions were applied at the initial stage, and the inhibitory effects of non-induced cells and persister cells on biofilm formation were observed after 12 h using a 96-well plate. The biomass of biofilms in the negative control group (100.00%) was significantly higher than in all treatment groups (antibiotic group: 53.24%; phage cocktail group: 55.17%; vB_KpnM_NB2 + antibiotic group: 43.03%; phage cocktail + antibiotic group: 40.00%), indicating that these interventions effectively inhibited biofilm formation by non-induced cells. Notably, the phage cocktail combined with antibiotics showed the strongest inhibitory effect (Fig. 8A). Consistent with the trend observed for normal cells, the biofilm formation rate of the persister cell treatment groups (antibiotic group: 72.78%; phage cocktail group: 73.91%; vB_KpnM_NB2 + antibiotic group: 73.91%; phage cocktail + antibiotic group: 66.16%) was lower than the negative control group (100.00%), suggesting that phage-based interventions can effectively inhibit biofilm formation in persister cells. However, as shown in the figure, persister cells exhibited reduced sensitivity to the same interventions, which may promote biofilm formation (Fig. 8B).

In the biofilm clearance experiment, mature biofilms formed by non-induced cells and persister cells were treated with the same interventions. The results showed that both phage cocktails and antibiotics had the ability to clear biofilms. In the non-induced group, the biofilm biomass in the negative control group remained almost unchanged, while all treatment groups showed a significant reduction in biofilm biomass. For example, after 12 h, the biofilm biomass in each group was as follows: antibiotic group 40.81%, phage cocktail group 38.12%, vB_KpnM_NB2 + antibiotic group 36.55%, and phage cocktail + antibiotic group 36.10% (Fig. 8C). In the persister cell group, the biofilm biomass in all treatment groups (antibiotic group 25.29%, phage cocktail group 21.29%, vB_KpnM_NB2 + antibiotic group 22.57%, phage cocktail + antibiotic group 21.71%) was significantly lower than in the negative control group (100%) after 12 h (Fig. 8D). No significant differences were observed among all treatment groups.

Overall, the study shows that both phage cocktail and antibiotic can inhibit and clear biofilm formation in *K. pneumoniae*, with the combination of both treatments being the most effective. While non-induced cells were more sensitive to these interventions, persister cells showed reduced sensitivity, suggesting that their metabolic state contributes to their resistance. Both phages and antibiotics significantly reduced biofilm biomass in mature biofilms formed by both non-induced cells and persister cells, highlighting the potential of phage and antibiotic in combating biofilm-related antibiotic resistance.

### CLSM observation of biofilms

To visually demonstrate the effects of the phage cocktail and amikacin on *K. pneumoniae* biofilms and to assess the impact of the phage cocktail and antibiotic on biofilm structure and bacterial viability, dynamic imaging was performed using CLSM in this study. As shown in Fig. 8E, the biofilm formation of *K. pneumoniae* under treatments with either the phage cocktail or the combined phage-antibiotic treatment was significantly reduced after 12, 24, 36, and 48 h, as evidenced by decreased fluorescence intensity. These results indicate that, in the absence of treatment, sensitive cells exhibit stronger proliferation and biofilm-forming ability compared to persister cells, whereas persister cells display typical low-metabolic and low-proliferative characteristics (Fig. 8F). The phage cocktail partially inhibited the proliferation and biofilm formation of *K. pneumoniae* XU1. More importantly, the combined treatment of the phage cocktail and antibiotics achieved nearly complete eradication and inhibition of biofilms in both sensitive and persister cells, demonstrating markedly superior efficacy compared to the use of the phage cocktail alone.

### Quantitative determination of CPS

**Fig 9:**
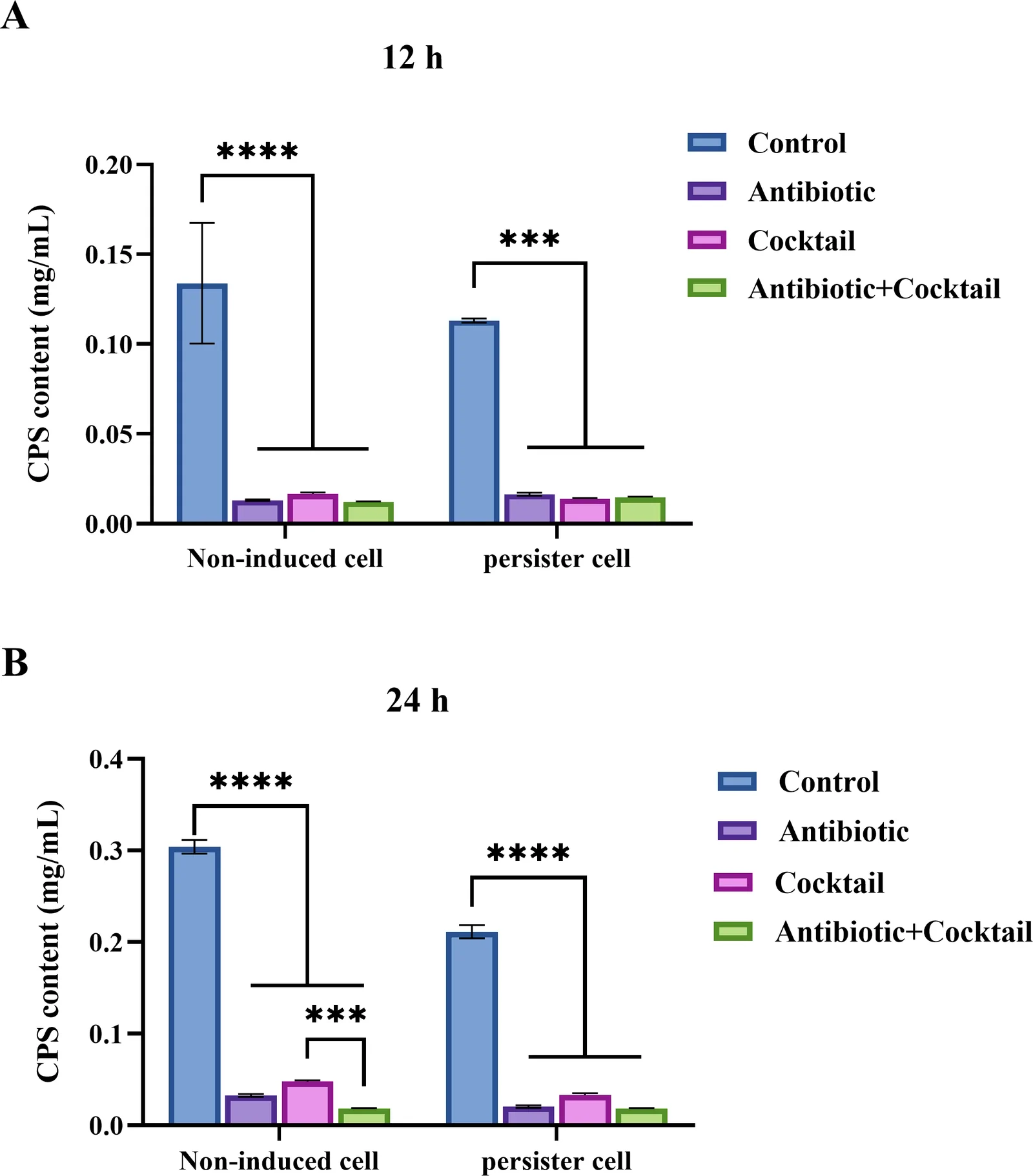
Quantification of CPS content. The CPS content was measured in non-induced cells and persister cells following treatment with antibiotics, bacteriophage cocktail, or their combination at 12 h (**A**) and 24 h (**B**). Data are presented as the mean ± standard deviation (SD) from three independent biological replicates. Statistical analysis was performed using one-way ANOVA followed by Dunnett’s post hoc test. ***P < 0.001, ****P < 0.0001.

Polysaccharide molecules exposed on the bacterial surface play a crucial role in phage adsorption (49). To evaluate the combined effect of the phage cocktail and antibiotics from the perspective of phage receptor binding, we quantified the CPS content in bacteria. The results are shown in Fig. 9; at both 12 and 24 h, the CPS content in non-induced cells and persister cells significantly increased with the extension of culture time. Interestingly, the CPS levels in the persister cells were significantly lower than those in the non-induced group. Compared to the control, treatments with antibiotics, phage cocktail, and their combination markedly suppressed CPS synthesis (*P* < 0.001 or *P* < 0.0001). For instance, at the 24-h time point, the combination therapy in persister cells showed a stronger inhibitory effect, with CPS content decreasing from 0.211 to 0.018 mg/mL, which was higher than any individual treatment (antibiotic: 0.020 mg/mL; phage cocktail: 0.033 mg/mL). These results suggest that the phage cocktail and amikacin act synergistically to inhibit CPS synthesis in *K. pneumoniae*, including persister cells, thereby weakening its outer membrane barrier.

## DISCUSSION

The emergence of multidrug-resistant *K. pneumoniae* and antibiotic-tolerant persister cells has become a major obstacle to effective clinical treatment (50). These persister cells, which exist in a metabolically dormant state and do not exhibit genetic resistance, are responsible for treatment failure, chronic infections, and disease recurrence (51, 52). Phage cocktails, by targeting multiple sites, complementing each other’s effects, and providing broad-spectrum coverage, have significantly enhanced antimicrobial efficacy and delayed the onset of resistance (53). In this study, three lytic phages, vB_KpnM_NB (1–3), were isolated, and a phage cocktail was formulated. The therapeutic effects of this phage cocktail, both alone and in combination with amikacin, on planktonic KPN, persister cells, and biofilms were evaluated. The results demonstrated that the phage cocktail, in combination with amikacin, synergistically eradicated persister cells, disrupted biofilms, and reduced CPS synthesis, thus providing a viable combined strategy for treating refractory KPN infections.

All three phages formed clear plaques with an icosahedral head and non-constrictive tail, consistent with the morphology of the Drexlerviridae family. Genomic analysis confirmed the absence of antibiotic resistance genes, virulence factors, integrases, repressors, and toxin-coding genes, verifying their strictly lytic life cycle and high biosafety for therapeutic use. The phylogenetic tree shows that the three phages share extremely high genetic homology. Thermal and pH stability tests indicated that the phages remained active under physiological conditions and showed rapid adsorption, supporting their potential application. The MOI for effective infection was relatively high, but significant lysis still occurred at lower MOIs, demonstrating their strong infectivity. Traditional phage replication kinetics generally suggest a positive correlation between the latent period and burst size, whereby a longer latent period provides more sufficient time for progeny phage assembly and consequently results in a larger burst size (54). In the present study, phages vB_KpnM_NB1 and vB_KpnM_NB2 exhibited relatively long latent periods (~100 min) but showed markedly different burst sizes (196.88 and 35.83 PFU/cell, respectively). Similar observations have been reported for *K. pneumoniae* phages. For example, phage KP1LMA exhibited a latent period of approximately 100 min with a relatively small burst size (7.9 ± 0.3 PFU/cell) (55), whereas phages LASTA and SJM3 showed latent periods of approximately 80 min with larger burst sizes (187 ± 37 and 155 ± 34 PFU/cell, respectively) (56). These findings indicate that the relationship between latent period and burst size is not strictly determined by replication duration alone. Previous studies have demonstrated that bacterial capsules can significantly influence phage adsorption and intracellular replication kinetics. The capsule may impede phage DNA injection and interfere with intracellular replication processes, thereby contributing to prolonged latent periods (57, 58). Furthermore, differences in the efficiency of phages in exploiting host resources may further alter the conventional relationship between latent period and burst size (59). The host range of the three phages was narrow and specific to KPN, minimizing off-target effects on commensal microbiota, supporting targeted antimicrobial intervention. Notably, phage vB_KpnM_NB3 exhibited a broader infective profile. This difference in host range is fundamentally attributed to functional divergence in the phage tail receptor-binding proteins (RBPs) and capsular depolymerases (60).

A stable KPN persister model was established using amikacin at 10× MIC. The model exhibited a typical biphasic killing curve, characteristic of persister cell formation, as described by Balaban et al. (39). In the model, susceptible cells were rapidly eliminated, followed by a plateau phase of surviving persister cells. Maffei et al. (61) also confirmed that persister cells are often in a low-metabolism dormant state, making them difficult to completely clear with conventional antibiotics. Our secondary exposure experiment confirmed that the revived persister cells remained sensitive to amikacin, ruling out genetic resistance factors and validating their persister phenotype. Compared to exponentially growing cells, persister cells exhibited significantly lower intracellular ATP levels, with delayed revival, explaining why traditional antibiotics often fail to clear persister cells—most drugs target the metabolic processes of actively growing cells (17, 18). Further optical microscopy observations showed that after antibiotic removal, persister cells slowly regained division capability, supporting their role as reservoirs for recurrent infections (62).

An interesting finding from *in vitro* killing assays was that the phage cocktail was more effective than individual phages in targeting both normal and persister cells. The cocktail achieved a 99.00% ± 1.59% clearance rate of persister cells, significantly higher than the 37.15%–50.67% clearance rate observed with individual phages. This superior performance is believed to stem from complementary receptor recognition mechanisms and delayed resistance development, which is consistent with findings in phage cocktail studies (63, 64). These results suggest that the phage cocktail effectively targets non-replicating persister cells, overcoming the limitations of single antibiotic therapies.

Biofilm formation is a key virulence factor for *Klebsiella* species and most other pathogenic bacteria (65). In *K. pneumoniae*, genetic regulation of the “biofilm switch” leads to reduced capsule production (41, 66). According to data from the NIH, biofilms are present in over 80% of bacterial infections, and biofilm formation is a key factor in the development of antibiotic resistance (41, 67). In this study, both the phage cocktail and amikacin inhibited biofilm formation and cleared established biofilms, with the combination showing the most significant effect. This finding is consistent with Ryan et al. (68), who showed that phage T4 combined with cefotaxime was more effective in inhibiting *E. coli* biofilm formation than cefotaxime alone. Biofilms derived from persister cells have denser structures and are less sensitive to single treatment methods, which corresponds to their role in chronic infections (69). Phages can degrade the polysaccharide components of biofilms, disrupt their dense structure, and enhance antibiotic permeability, thereby synergistically eliminating persister cells and inhibiting biofilm formation (70). CLSM confirmed that combination therapy almost completely eliminated both the biofilm structure and activity, while single treatments only partially reduced the biomass. These results support the use of phage-antibiotic combination therapies to overcome the physical and physiological barriers of biofilms.

CPS is a key virulence determinant and phage adsorption barrier for KPN (71). In this study, CPS levels in persister cells at 12 and 24 h were lower than in non-induced cells, which is associated with reduced extracellular matrix synthesis due to their dormant metabolic state (17). Notably, the phage cocktail combined with amikacin significantly suppressed CPS production, lowering CPS levels more than either treatment alone. Consistent with our findings, recent studies have shown that phage depolymerases or phage therapy can degrade or inhibit bacterial capsule production (72, 73). By reducing CPS levels, this combination therapy enhances phage adsorption, weakens the bacteria’s immune evasion mechanisms, and disrupts outer membrane integrity. In contrast, antibiotic monotherapy may induce compensatory capsule upregulation as a stress response (74). The dual reduction in bacterial survival and CPS levels is a key mechanism underlying the synergistic effect of the phage cocktail and amikacin.

Our study is not without limitations. Although the *in vitro* experimental results are promising, it must be acknowledged that several limitations remain that need to be addressed in future research. The precise host receptors for these phages, as well as the detailed molecular pathways mediating the synergistic effects of the phage cocktail and amikacin against persister bacteria and biofilms, have yet to be fully elucidated. All efficacy assessments were conducted under *in vitro* conditions, which cannot fully replicate the complexity of the *in vivo* microenvironment, including host immune responses, blood circulation, phage distribution, and physiological clearance mechanisms. The significant variation of phage therapy lies in its pharmacokinetics and pharmacodynamics *in vivo* (75), and the actual therapeutic effects of phage-antibiotic cocktail therapy in animal models or clinical patients still need to be validated. There is a lack of evidence regarding its efficacy in preclinical and clinical studies (76). In addition, this study did not systematically evaluate the long-term safety, the potential immunogenicity of phage particles, or the risk of phage resistance evolution during prolonged application (75, 77).

In summary, phage vB_KpnM_NB cocktail, synergizing with amikacin, exhibits significant antimicrobial activity against planktonic *K. pneumoniae*, persister cells, and biofilms. This phage cocktail is safe, stable, and highly targeted, with its combination strategy effectively addressing the issue of antibiotic inefficacy against dormant and biofilm-encapsulated bacterial strains. These findings provide a promising foundation for the translational application of phage-antibiotic combination therapy in the treatment of refractory *K. pneumoniae* infections. Future research will focus on *in vivo* efficacy evaluation in animal models, mechanism analysis based on transcriptomics and metabolomics, and optimization of the phage cocktail formulation to enhance clinical applicability.

### Conclusion

The results of this study indicate that the combination of phage therapy and amikacin significantly reduces the population of *K. pneumoniae* persister cells. This combination therapy not only decreases bacterial biofilm formation but also reduces the production of capsular polysaccharides. This strategy offers a promising alternative for combating antibiotic-persistent bacterial infections.

## Data Availability

All data supporting the results of this study are available within the paper. The sequencing data of phages vB_KpnM_NB1, vB_KpnM_NB2, and vB_KpnM_NB3 have been deposited in the GenBank database at the National Center for Biotechnology Information (NCBI) under the accession numbers PZ440473, PZ440474, and PZ454251, respectively.
